# Radiosynthesis and Preclinical Evaluation of ^11^C-VA426, a Cyclooxygenase-2 Selective Ligand

**DOI:** 10.1155/2019/5823261

**Published:** 2019-09-24

**Authors:** Assunta Carpinelli, Paolo Rainone, Sara Belloli, Annalisa Reale, Andrea Cappelli, Giuliani Germano, Valentina Murtaj, Angela Coliva, Giuseppe Di Grigoli, Angela Valeri, Maria Carla Gilardi, Luigi Gianolli, Maurizio Anzini, Rosa Maria Moresco

**Affiliations:** ^1^Institute of Molecular Bioimaging and Physiology of CNR, 20090 Segrate, Italy; ^2^PET and Nuclear Medicine Unit, San Raffaele Scientific Institute, 20132 Milan, Italy; ^3^Doctorate School of Molecular and Translational Medicine, University of Milan, 20122 Milan, Italy; ^4^Department of Biotechnology, Chemistry, and Pharmacy, DoE 2018–2022, University of Siena, 53100 Siena, Italy; ^5^PhD Program in Neuroscience, Department of Medicine and Surgery, University of Milano-Bicocca, 20900 Monza, Italy; ^6^Department of Medicine and Surgery, University of Milano-Bicocca, 20900 Monza, Italy

## Abstract

Cyclooxygenase-2 (COX-2) is involved in the inflammatory response, and its recurrent overexpression in cancers as well as in neurodegenerative disorders has made it an important target for therapy. For this reason, noninvasive imaging of COX-2 expression may represent an important diagnostic tool. In this work, a COX-2 inhibitor analogue, VA426 [1-(4-fluorophenyl)-3-(2-methoxyethyl)-2-methyl-5-(4-(methylsulfonil)phenyl)-1*H*-pyrrole], was synthesized and radiolabelled with the ^11^C radioisotope. The ex vivo biodistribution profile of ^11^C-VA426 was evaluated in the brain and periphery of healthy rats and mice and in brain and periphery of inflammation models, based on the administration of LPS. ^11^C-VA426 synthesis with the *t*BuOK base showed optimal radiochemical yield (15 ± 2%) based on triflate activity, molar activity (range 37–148 GBq/*μ*mol), and radiochemical purity (>95%). Ex vivo biodistribution studies showed a fast uptake of radioactivity but a rapid washout, except in regions expressing COX-2 (lungs, liver, and kidney) both in rats and in mice, with maximum values at 30 and 10 minutes p.i., respectively. LPS administration did not show significant effect on radioactivity accumulation. Celecoxib competition experiments performed in rats and mice treated with LPS produced a general target unrelated reduction of radioactivity concentration in all peripheral tissues and brain areas examined. Finally, in agreement with the negative results obtained from biodistribution experiments, radiometabolites analysis revealed that ^11^C-VA426 is highly unstable in vivo. This study indicates that the compound ^11^C-VA426 is not currently suitable to be used as radiopharmaceutical for PET imaging. This family of compounds needs further implementation in order to improve in vivo stability.

## 1. Introduction

Cyclooxygenase (COX) is the triggering enzyme for the conversion of arachidonic acid to prostaglandins, and two isoforms (COX-1 and COX-2) have been identified and studied [1]. COX-1 is known as the ubiquitous isoform, which is constitutively expressed, while COX-2 is poorly expressed in normal conditions and is therefore undetectable in most tissues except in the kidney, intestine, lung, liver, heart, and brain [2, 3]. Nevertheless, COX-2 is rapidly induced in response to various inflammatory stimuli, hormones, and growth factors and has consequently been referred as the “inducible” isoform [4]. Genetic and pharmacological studies in rodents suggest that both isoforms might be important in maintaining physiological homeostasis and contribute to the inflammatory response, and it has been shown that selective COX-2 inhibition contributes to anti-inflammatory processes while COX-1 inhibition is involved in the onset of side effects [5, 6]. It is widely accepted that deregulation of COX-2 expression plays a key role in tumour progression [7, 8] and in the development of chronic inflammation related pathological conditions such as rheumatoid arthritis or neurodegenerative diseases including Parkinson and Alzheimer's disease [9, 10]. Furthermore, recent reports indicate that the basal expression of COX-2 is important for the maintenance of the physiology of several organs such as the kidney, heart, and brain [11, 12]. Thus, the noninvasive evaluation of COX-2 distribution in all body regions in nonpathological and pathological conditions seems to be crucial to understand the involvement of this key enzyme in the inflammation response and in homeostasis preservation. Moreover, the possibility to measure COX-2 expression in vivo may provide a suitable biomarker for disease staging and therapy evaluation [13].

Positron emission tomography (PET) is a functional imaging technique that is used in nuclear medicine to follow metabolic processes in vivo for the diagnosis and staging of different pathologies, such as cancer and brain disorders [14]. By taking advantage of specific radiopharmaceuticals designed either to follow several molecular pathways in vivo or to bind to different receptor subtypes, it is possible to study several biological features, including inflammation [15]. Noninvasive PET imaging of COX-2 expression might provide a better understanding of chronic inflammation in vivo, which is associated with the progression of most cancers and neurodegenerative diseases [16]. A large number of radiolabelled COX-2 inhibitors, most of them based on the celecoxib backbone [17], have been developed and tested especially on tumour-associated inflammation in rodents. However, these studies exhibited several limitations for in vivo imaging application [18].

In this work, a selective COX-2 inhibitor, VA426 [1-(4-fluorophenyl)-3-(2-methoxyethyl)-2-methyl-5-(4-(methylsulfonyl)phenyl)-1*H*-pyrrole], has been synthesized and labelled with the radioisotope ^11^C. In a previous study [19], VA426 showed a high affinity for the active site of COX-2 in vitro (IC_50_ = 0.018 *μ*M, IC_50_ COX-1/IC_50_ COX-2, Selectivity Index >5555), without toxic effects. Therefore, our aim was to optimize and validate a fast and fully automated method to produce ^11^C-VA426 with high yields and optimal molar activity and to evaluate the in vivo/ex vivo biodistribution, specificity, and stability in healthy and inflammation animal models.

## 2. Materials and Methods

### 2.1. Chemicals, Reagents, and Radiosynthesis Instrumentation

The precursor [VA425], 1-((4-fluoro)phenyl)-3-2-hydroxyethyl)-2-methyl-5-methylsulfonyl)phenyl-1*H*-pyrrole, and the reference compound [VA426] 1-(4-Fluorophenyl)-3-(2-methoxyethyl)-2-methyl-5-(4-methyl-sulfonyl)phenyl)-1*H*-pyrrole were previously described, synthetized, and kindly provided by prof. Anzini (University of Siena) [19]. Helium and hydrogen purifier were supplied by W.R Grace (Columbia, Maryland, USA). Sublimated iodine, NaH 60%, *t*BuOK (>98%), AcONH_4_ (98%), AgOTf (>99.95%), diethylether, DMSO (>99.95%), MeCN hypersolv for HPLC (99.7%), acetone >99.5%, absolute EtOH, and Carbopack-TM adsorbent 80–100 mesh (Supelco) were purchased from Sigma-Aldrich (Saint Louis, MO, USA). All analytical grade chemicals and solvents were used without further purification, and reagents and instrumentation mentioned below were purchased according to a previous study performed by Coliva et al. [20]. Loading target gas N_2_ (0.5% O_2_) was supplied by Gruppo SAPIO. The Ni catalyst (Shimalite Ni reduced, 80/100 mesh) was purchased from Shimadzu and NaOH 1 M by Fluka. The powder of silver triflate impregnated carbon was prepared in our laboratory dissolving 1 g of silver trifluoromethanesulfonate in 150 mL of anhydrous diethyl ether. To this solution, 2 g of Graphpack C (80/100 mesh) was added and the suspension was stirred under reduced pressure in the dark to allow the ether to dry off slowly. Once the ether was evaporated, the resulting powder was dried for at least 2 hours under vacuum (0.26 mbar). The dry powder was stored into a dark glass bottle. Sterile ethanol, water, and saline 0.9% for final formulation were supplied by S.A.L.F. Sep-Pak tC18 plus light cartridges (130 mg) were purchased from Waters. Millex-GV filters were obtained from Millipore. Semipreparative ACE-C18, 5 *μ*m, 250 × 10 mm, and analytical ACE C18, 5 *μ*m, 250 × 4.8 mm, HPLC columns were supplied by Agilent. The automatic synthesis module TracerLab FX-C Pro and the remote control software were purchased from General Electric Medical System. Semipreparative HPLC equipment was a Sykam pump S1021. Analytical HPLC consists of a 515 pump, a 486 tunable absorbance UV detector (operated at 254 nm), and a NaI-radio-detector (Flowcount FC 3200 Eckert & Ziegler Radiopharma). Radionuclide identification was performed using a NaI (Tl) gamma detector 3M3/3-X Ortec, Advanced Measurement Technology.

### 2.2. Preparation of the Synthesis Module

**Figure 1:**
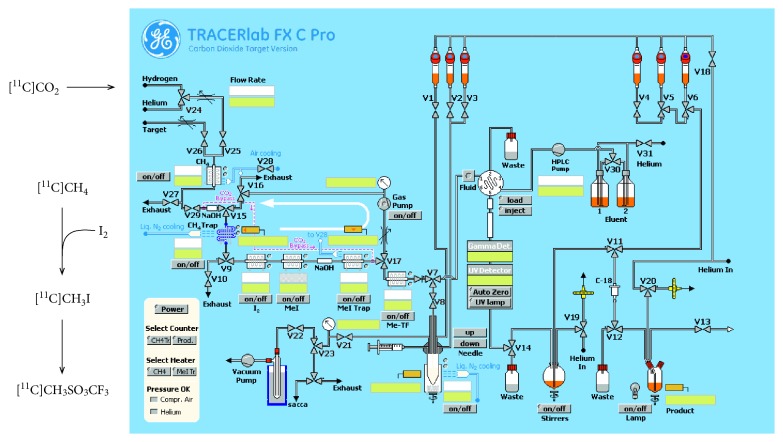
Synoptic module setup of the commercial automated synthesizer (TracerLab FxC-Pro). CO_2_ bypass was not used for the synthesis of ^11^C-VA426.

The synoptic synthesis module is represented in Figure 1. All glassware and tubing of the module were rinsed with pure water, acetone, or ethanol and then dried using a stream of helium. During the preparation of the synthesis, vessels were filled as follows:

- Vial 1 (valve V1): HPLC eluent to quench the reaction and dilute before HPLC injection
- Vial 4 (valve V4): water for washing the SPE cartridge
- Vial 5 (valve V5): absolute ethanol for elution of the SPE cartridge
- Vial 6 (valve V6): sterile saline for final rinsing of the SPE cartridge and dilution of the eluate
- Round bottom flask: sterile water for dilution of the HPLC fraction after peak cutting and pH adjustment

A tC18 cartridge was conditioned with 5 mL of ethanol followed by 20 mL of sterile water, dried, and connected to its dedicated position. Vials 4, 5, and 6, the round bottom flask and the SPE cartridges were used for formulation. The reactor was filled with 3.2 mg of precursor (VA425) and 1.5 mg of *t*BuOK dissolved in 0.150 mL of DMSO few minutes before starting the synthesis and then placed into the heating block. The AgOTf-oven contained a tube filled with about 500 mg of graphitized carbon impregnated with AgOTf. MeI trap-oven contained about 300 mg of Porapak Q.

### 2.3. Synthesis of [^11^C]MeOTf

The synthesis of [^11^C]methyl triflate ([^11^C]MeOTf) was performed according to the method used in a recent study by Coliva et al. [20], in which the authors set up the radiosynthesis of an amyloid-plaques specific radiotracer, ^11^C-PIB, by methylation of its precursor. Briefly, [^11^C]MeOTf was generated by reaction of [^11^C]CH_3_I with AgOTf in an online flow-through process at 200°C under helium gas flow. The production of [^11^C]MeOTf is completed in 180 seconds with a radiochemical yield of about 55–60% (n.d.c). The process was completely automated using the software GE, 2.2.1 version, and all the procedures are schematized in Figure 1.

### 2.4. Radiosynthesis of ^11^C-VA426

**Figure 2:**
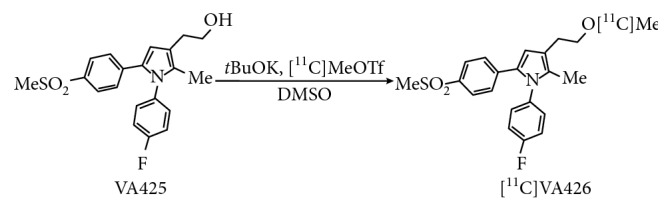
Synthesis scheme of ^11^C-VA426 by [^11^C]MeOTf approach.

[^11^C]MeOTf was bubbled, with a flow rate of 25 mL/min at room temperature, into the reactor containing the precursor VA425 (3.2 mg, 8.6 *μ*mol dissolved in 150 *μ*L DMSO) and *t*BuOK (1.5 mg, 13 *μ*mol), in order to obtain the labelled compound ^11^C-VA426, as presented in Figure 2. The reactor was then heated at 80°C for 2 minutes, and the reaction was quenched by adding the HPLC mobile phase (1.4 mL). This solution was transferred via fluid detector into the HPLC loop and injected automatically.

### 2.5. Purification of ^11^C-VA426

**Figure 3:**
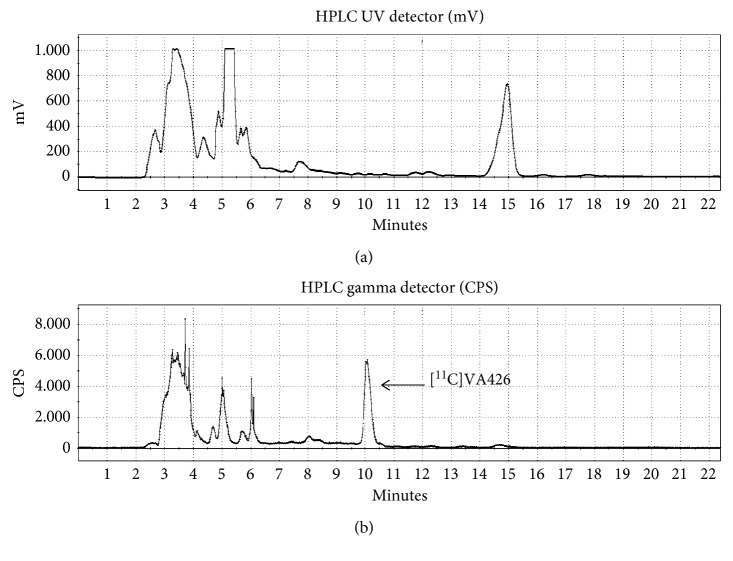
Semipreparative HPLC purification of ^11^C-VA426 radiolabelling.

The crude mixture was purified by semipreparative HPLC. The apparatus was equipped with an UV detector (*λ* = 280 nm) and a Geiger–Muller tube for detection of radioactivity placed in series. The product was eluted on the C18 ACE column with CH_3_CN/AcONH_4_ 0.05 M (60/40, v/v) as mobile phase and a flow rate of 5 mL/min. The products eluted as follows (Figure 3):

- Precursor VA425 RT_1_ = 5 minutes
- Product ^11^C-VA426 RT_2_ = 10 minutes

The ^11^C-VA426 fraction was collected and diluted with 30 mL of sterile water. To change the solvent and to concentrate the product, this solution was then loaded on a tC18 SPE cartridge. After washing with 10 mL of sterile water, the product was recovered with 1.2 mL of absolute ethanol followed by 8.8 mL of saline. The final solution (total volume 10 mL) was then transferred to a shielded laminar air-flow hot cell and, there, filtered through a Millex-GV filter (0.22 *μ*m) into a sterile vial.

### 2.6. Quality Control of ^11^C-VA426

**Figure 4:**
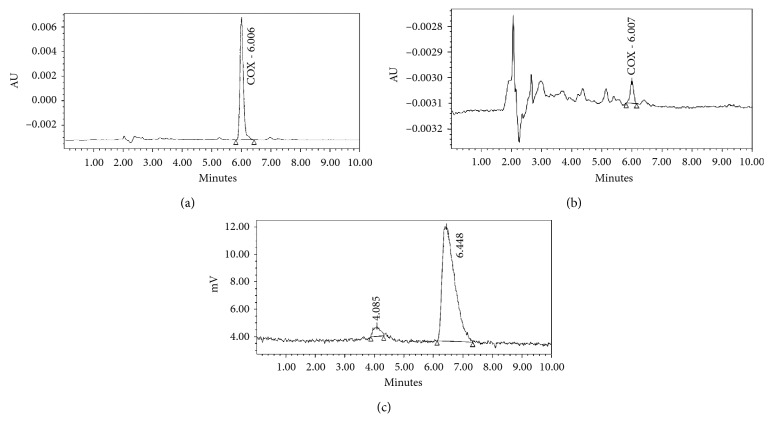
Analytical HPLC quality control. (a) UV chromatogram of the reference standard VA426; (b) UV chromatogram of ^11^C-VA426; (c) radiochromatogram of ^11^C-VA426 showing a radiochemical purity ≥95%.

Chemical and radiochemical purities of the labelled compound ^11^C-VA426 were determined by analytical HPLC (Eckert & Ziegler Radiopharma Inc. Hopkinton, MA, USA). The instrument was equipped with a UV detector (*λ* = 254 nm) coupled to a radioactivity flow detector. Separation was achieved on the C18 ACE column with CH_3_CN/AcONH_4_ 0.025 M (70/30; v/v) as the mobile phase and at a flow rate of 1 mL/min. The products eluted as follows (Figure 4):

- Precursor VA425 RT_1_ = 4 minutes
- Product ^11^C-VA426 RT_2_ = 6.5 minutes

The quantification of ^11^C-VA426 was developed using HPLC for comparison with a standard at a known concentration. The radiochemical purity was calculated as the percentage of the total radioactivity related to ^11^C-VA426.

### 2.7. Animals

Adult male Sprague Dawley (SD) rats (250–300 g, Envigo RMS, Italy) and male CD-1 mice (35–45 g, Envigo RMS, Italy) were used for this study. Animals were maintained and handled in compliance with the institutional guidelines for the care and use of experimental animals (IACUC) of San Raffaele Institute (Milan, Italy), which have been notified to the Italian Ministry of Health and approved by the Ethics Committee of the San Raffaele Scientific Institute (Study no. 722/2016-PR).

### 2.8. Biodistribution Kinetic Profile in Healthy Rats

Nine SD rats were injected through the tail vein with 9.25 ± 2.5 MBq of ^11^C-VA426 and euthanized under general anaesthesia, with a mixture of 4% isoflurane in air, at different time points (10, 30, and 60 minutes) from injection (*n* = 3 per time). Blood was collected by retro-orbital sampling immediately before the sacrifice. Plasma was separated by centrifugation, and 100 *μ*L of blood and plasma were counted in a *γ*-counter (LKB Compugamma CS 1282). Peripheral organs (heart, lung, liver, intestine, kidney, testis, and muscle) and brain regions (frontal, right and left cortex, right and left striatum, hippocampus, thalamus, hypothalamus, pons, and cerebellum) were immediately removed and rinsed in cold saline solution. Each sample was then placed in a test tube and weighed, and the radioactivity was measured using a *γ*-counter (LKB Compugamma CS 1282). An additional aliquot (0.1 mL) of radioactive solution was diluted to 1 : 10, 1 : 100, and 1 : 1000 and used to calculate the standard curve. Radioactivity concentration was calculated as the percentage of injected dose per gram of tissue (%ID/g).

### 2.9. Specificity Study in Healthy Rats

In order to evaluate the radiotracer binding specificity to COX-2 in healthy rats, a competition study was performed. Two minutes before the intravenous injection of ^11^C-VA426, four animals were pretreated with the COX-2 specific inhibitor celecoxib (Sigma-Aldrich, Italy) (10 mg/kg, i.v.) [21] and four animals with vehicle solution alone (100 *μ*l of DMSO, Sigma-Aldrich) [22]. All rats were then injected intravenously with 9.25 ± 2.7 MBq of ^11^C-VA426 and sacrificed at the time of maximum uptake (30 minutes p.i.), previously assessed in the kinetic study. Central and peripheral samples were dissected as described above and counted, following the same protocol used for the biodistribution kinetic study. Radioactivity concentration in samples was calculated as %ID/g.

### 2.10. Biodistribution Kinetic Profile in a Brain Inflammation Rat Model

In order to trigger nigrostriatal inflammation, eighteen SD rats were anesthetized (zoletil, 25 mg/kg, i.p.) and stereotaxically injected into the right striatum with 3 *μ*L (3.33 *μ*g/*μ*l) of lipopolysaccharide (LPS, Sigma-Aldrich, Italy) [23, 24] and in the contralateral hemisphere with saline (negative control) at the following coordinates: *A* = +0.5, *L* = ±3.0, and *V* = –5.0 mm. One day after LPS injection, the first experimental group (*n* = 9) was injected through the tail vein with 9.25 ± 2.2 MBq of ^11^C-VA426, and three rats per time point (10, 30, and 60 minutes) were euthanized under general anaesthesia, with a mixture of 4% isoflurane in air. Central and peripheral samples were dissected as described above and counted, following the protocol described for the biodistribution kinetic study. Twelve days after LPS injection [25], the second group of animals (*n* = 9) underwent the same protocol. Radioactivity concentration in samples was calculated as %ID/g.

### 2.11. Biodistribution Kinetic Profile in Healthy Mice

Male CD-1 mice were injected through the tail vein with 5.3 ± 1.4 MBq of ^11^C-VA426 and sacrificed after 10, 30, and 60 minutes (*n* = 3 per time point) under general anaesthesia with a mixture of 4% isoflurane in air. Blood was collected by retro-orbital sampling immediately before sacrifice. Plasma was separated by centrifugation, and 100 *μ*L of blood and plasma were counted in a *γ*-counter (LKB Compugamma CS 1282). Different peripheral organs (heart, lung, liver, intestine, stomach, spleen, kidney, testis, and muscle) and brain regions (cortex, striatum, hippocampus, thalamus, hypothalamus, pons, substantia nigra, and cerebellum) were immediately sampled, rinsed with cold saline, and placed in preweighed tubes for counting. Radioactivity concentration was calculated as %ID/g.

### 2.12. PET Kinetic Study in Healthy Mice

Two CD-1 mice were anaesthetized with a mixture of 4% isoflurane in air for the imaging with the YAP-(S)PET system (ISE Srl). Each mouse was placed in a prone position on the PET scanner bed with the abdomen centred in the field of view (FOV). Five minutes before the intravenous injection of ^11^C-VA426, one mouse was pretreated with the COX-2 specific inhibitor celecoxib (10 mg/kg, i.v.) [21] in vehicle (100 *μ*l of DMSO, mouse 1) and the other with vehicle alone (mouse 2) [22]. Mouse 1 was then injected intravenously with 1.85 MBq of ^11^C-VA426 and mouse 2 with 3.7 MBq, and dynamic PET data were acquired for 30 minutes, according to the following schedule: four scans of 2.5 minutes followed by four of 5 minutes. PET data were acquired in list mode, using the full axial acceptance angle of the scanner (3D mode) and then reconstructed with the expectation maximization (EM) algorithm. All images were calibrated with a dedicated phantom, corrected for the radionuclide half-life decay, and quantified as ID/g.

### 2.13. Specificity Evaluation in a Peripheral Inflammation Mouse Model

Specificity evaluation studies of ^11^C-VA426 were performed on a murine model of peripheral inflammation, after COX-2 specific inhibitor administration. Six hours before the study, six mice received intraperitoneal injection of lipopolysaccharide (10 mg/Kg) [26, 27], in order to promote peripheral inflammation response. Three of the LPS-treated mice were injected i.v. with 10 mg/kg of celecoxib, five minutes before the ^11^C-VA426 injection, to carry out a competition assay for the COX-2 binding sites. All mice were sacrificed at the time of maximum uptake (10 minutes p.i.), previously assessed in healthy mice, after 3.9 ± 1.1 MBq injection of the radiotracer. Peripheral samples were dissected and counted, as well as for biodistribution studies. Radioactivity concentration in samples was calculated as %ID/g.

### 2.14. Stability Study in Healthy Mice

Mice (*n* = 3) were injected through the tail vein with 3 ± 0.9 MBq of ^11^C-VA426 and sacrificed 10 minutes later, under general anaesthesia. Blood was collected by retro-orbital sampling and processed to obtain plasma, as described above. Radioactive species contained in plasma were extracted by mixing an aliquot (500 *μ*L) of plasma with CH_3_CN solution (1 : 1 v/v). Acetonitrile extracts were centrifuged and supernatant filtered with a MillexGX® syringe system for HPLC (Gilson Italia Srl, 321 pump, UV/VIS-151) injection. For metabolites analysis in liver, a sample was placed in a tissue homogenizer (Potter-Elvehjem) with 2 mL of saline, mechanically sheared until complete homogenization and subsequently treated as described for plasma. HPLC analyses were performed at room temperature using a C_18_, 250 × 10 mm, 5 *μ*m column, CH_3_CN/AcONH_4_ 0.05 M (60/40, v/v) as mobile phase, a flow rate of 4.5 mL/min, and UV = 254 nm. Eluted fractions were collected every 30 seconds, for a total of 14 minutes, and counted with a *γ*-counter (LKB Compugamma CS 1282).

### 2.15. Statistical Analysis

Values are expressed as mean ± SEM. The statistical significance of differences between groups was evaluated with unpaired Student's *t*-test, while among different brain areas of the same subject, with paired Student's *t*-test. A *p* value lower than 0.05 was considered significant.

## 3. Results

### 3.1. Radiosynthesis of ^11^C-VA426

Different bases were tested for the deprotonation of the VA425 hydroxyl group. *t*BuOK was more efficient than NaH, even though the latter was the strongest base tested, as presented in Table 1. Furthermore, as expected, aqueous 1 M NaOH gave poor radiochemical yield of the target product and some other by-product of MeSO_2_ methylation, even when a substoichiometric amount was employed (0.37 mol/mol of VA425). Synthesis time, elapsed from trapping of methane on CH_4_ trap to collection of the final compound, was about 40 minutes. Radiochemical yields of ^11^C-VA426 (Table 1) are calculated as the fraction of the activity, not decay corrected, related to the product using [^11^C]MeOTf activity as starting value. The best result was reached using *t*BuOK as base, with 15 ± 2% of yield and a radiochemical purity >95%. Finally, molar activity of the tracer was very high, in the range of 37–148 GBq/*μ*mol.

### 3.2. Biodistribution Kinetic Profile in Healthy Rats

**Figure 5:**
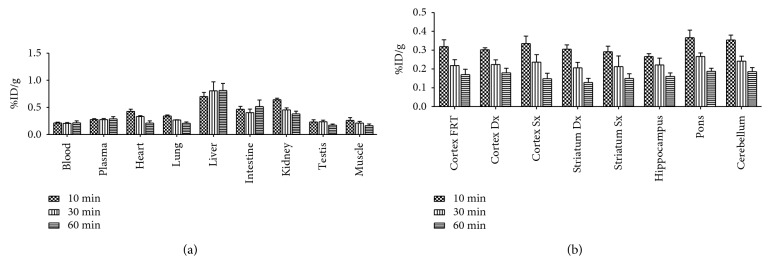
Ex vivo biodistribution of ^11^C-VA426 (a) in the periphery and (b) in the brain of healthy rats (*n* = 9, three per time point). Uptake values in sampled peripheral areas are expressed as %ID/g.

A biodistribution study was performed in three rats per time point (10, 30, and 60 minutes), to assess the kinetic profile of the ^11^C-VA426 uptake. Considering peripheral sampled tissues, the radiotracer rapidly accumulated in the liver (0.81 ± 0.28 %ID/g, at 30 min p.i.) and intestine, remaining stable over time. In other tissues [28] such as the kidney, heart, and lung, ^11^C-VA426 reached highest uptake values at 10 minutes p.i. and cleared thereafter (Figure 5(a)). The concentration of ^11^C-VA426 within different brain regions was comparable, at the different time points. Also in brain, radioactivity concentration reached the highest values at 10 min p.i., slowly decreasing thereafter (Figure 5(b)). For this reason, 30 minutes posttracer injection was selected as the optimal time point for further tracer characterization experiments in rats.

### 3.3. Specificity Study in Healthy Rats

**Figure 6:**
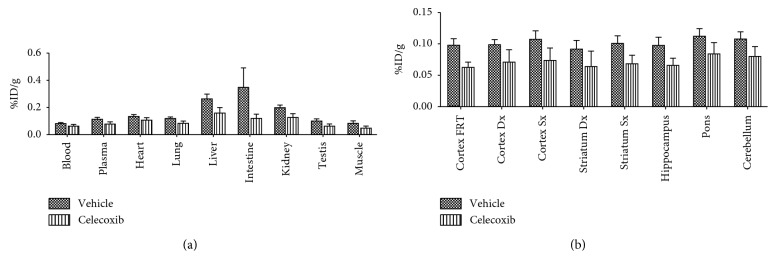
Inhibition study in healthy rats (*n* = 6, three per group) was performed after administration of celecoxib, a COX-2 specific inhibitor, or vehicle DMSO (control group). Ex vivo biodistribution at 30 minutes after ^11^C-VA426 injection (a) in the periphery and (b) in the brain. Uptake values are expressed as %ID/g.

^11^C-VA426 radiotracer specificity was evaluated using the COX-2 inhibitor celecoxib (10 mg/kg), as competitor. Celecoxib pretreated rats showed in all peripheral tissues (Figure 6(a)) and central (Figure 6(b)) areas a reduction of uptake values, slightly higher in the intestine, liver, and kidney (−39%, −65%, and −36%, respectively). Nevertheless, the results obtained from the study showed no significant differences between the vehicle and celecoxib pretreated rats.

### 3.4. Biodistribution Kinetic Profile in a Brain Inflammation Model

**Figure 7:**
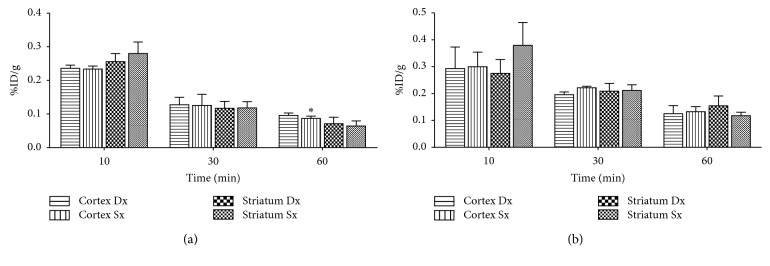
Ex vivo biodistribution of ^11^C-VA426 in the brain inflammation model; neuroinflammation was induced by intracranial LPS administration into the right nigrostriatal region (PBS on the left, as control), on two experimental groups (*n* = 9 per group). The first group, LPS 1d, was injected with ^11^C-VA426 one day after LPS treatment (*n* = 3 per time point) and the second group, LPS 12d, twelve days after LPS treatment (*n* = 3 per time point). Uptake values in brain areas are expressed as percentage of injected dose per gram of tissue (%ID/g) (^*∗*^*p* < 0.05 vs. right cortex, for paired Student's *t*-test).

COX-2 is minimally constitutively expressed in basal condition. For this reason, ^11^C-VA426 was examined after a LPS administration. Neuroinflammation was induced on two sets by nine SD rats through intracranial LPS administration into the right nigrostriatal region (PBS on left, as control). The first set of animals underwent the biodistribution study one day after toxin injection, in order to evaluate the radiotracer uptake in LPS-lesioned areas compared to the healthy contralateral hemisphere. In striatal regions, no significant differences of ^11^C-VA426 concentration were observed between the lesioned hemisphere and the contralateral PBS-injected control (Figure 7, LPS 1d) at different experimental times (10, 30, and 60 minutes, *n* = 3 per time point). Cortex exhibited the same trend, although at the latest time point (60 min p.i.), the right LPS-lesioned cortex showed a significant but negligible increase of tracer uptake when compared to the contralateral healthy region (0.096 ± 0.012 and 0.087 ± 0.012, respectively; *p* < 0.05). Twelve days after LPS injection (Figure 7, LPS 12d), no differences were found in the second set of animals between LPS-treated and healthy contralateral hemisphere, in all the regions analysed.

### 3.5. Biodistribution Kinetic Profile in Healthy Mice

**Figure 8:**
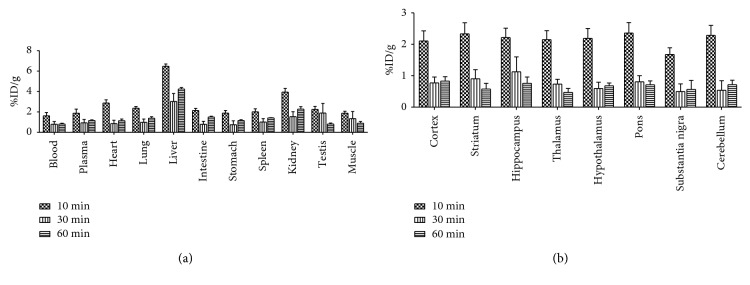
Ex vivo biodistribution of ^11^C-VA426 (a) in the periphery and (b) in the brain of healthy mice (*n* = 9, three per time point). Uptake values in sampled peripheral areas are expressed as percentage of injected dose per gram of tissue (%ID/g).

In healthy mice, ^11^C-VA426 accumulated mainly in the liver, showing maximum uptake values (7.72 ± 2.52 %ID/g) at 10 minutes after injection (Figure 8(a)), followed by the kidney, intestine, lung, and heart, which are considered as specific COX-2 expressing regions. Lower levels of uptake were observed in the remaining tissues. At latest time (60 minutes p.i.), radioactivity concentration decreased in all the regions examined. As observed in periphery, all brain regions reached the maximum values of radioactivity concentration at 10 minutes after tracer injection (2.69 ± 0.81%ID/g in pons), decreasing thereafter (Figure 8(b)). In central areas, no selective uptake region was observed during the entire experimental frame.

### 3.6. In Vivo PET Explorative Study in Healthy Mice

**Figure 9:**
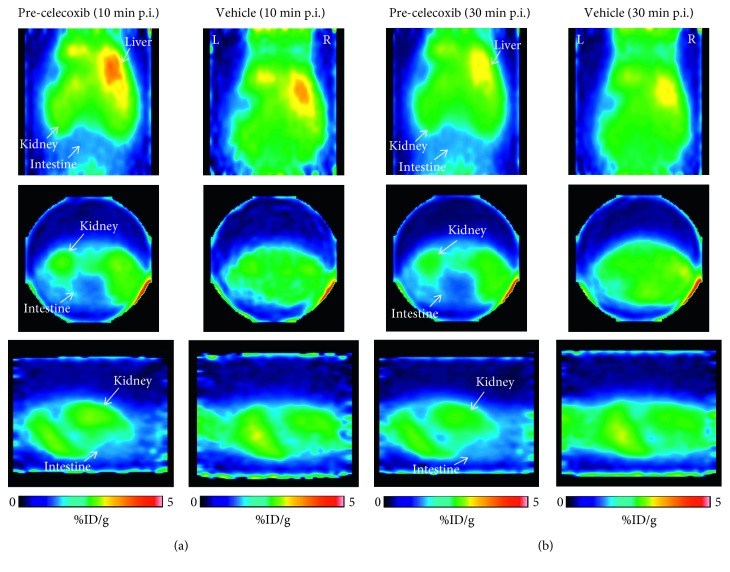
PET images of ^11^C-VA426 distribution in mouse (a) at 10 and (b) 30 minutes after injection. Each acquisition was shown in coronal, transaxial, and sagittal sections, respectively. Mice were anaesthetized with a mixture of 2% isoflurane in air, injected intravenously with 1.85 MBq (mouse 1) and 3.7 MBq (mouse 2) of ^11^C-VA426 and, respectively, pretreated (5 min before) with celecoxib or vehicle (DMSO). Dynamic PET data were acquired for 30 minutes (four scans of 2.5 minutes followed by four of 5 minutes). White arrow indicates the main anatomical regions of ^11^C-VA426 distribution. In coronal views, R (right) and L (left) indicate the spatial orientation of mouse.

We performed a preliminary in vivo PET kinetic evaluation of ^11^C-VA426 biodistribution, in two healthy mice (celecoxib preinjected or vehicle as control). PET images examined from 0 to 30 minutes after ^11^C-VA426 injection showed that radioactivity accumulates rapidly and primarily in the liver and kidneys (Figure 9(a)), slightly decreasing at 30 minutes (Figure 9(b)). Celecoxib pretreatment slightly reduced radioactivity distribution in intestine regions, at both times, as shown in Figures 9(a) and 9(b) (on the left).

### 3.7. Specificity Study in Peripheral Inflammation Model

**Figure 10:**
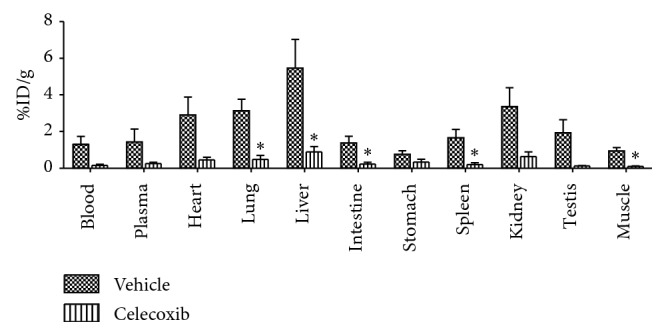
Inhibition study in the peripheral inflammation model; inflammation was induced by intraperitoneal injection of LPS six hours before the study. Mice were treated with celecoxib (*n* = 3) or vehicle (*n* = 3) prior ^11^C-VA426 injection and sacrificed after 10 minutes. Uptake values in peripheral areas are expressed as percentage of injected dose per gram of tissue (%ID/g) (^*∗*^*p* < 0.05 vs. vehicle).

A competition study was performed in a model of peripheral inflammation (systemic LPS-injection) by celecoxib preinjection. In this study, mice were subdivided in two groups: LPS-treated mice (*n* = 3, controls) and LPS-treated mice plus celecoxib administration (*n* = 3). As reported in Figure 10, mice celecoxib induced a dramatic reduction in tracer uptake (%ID/g), in all peripheral regions examined, including plasma, indicating that the effect was not associated with the competition at COX-2 binding site.

### 3.8. Analysis of Metabolites

**Figure 11:**
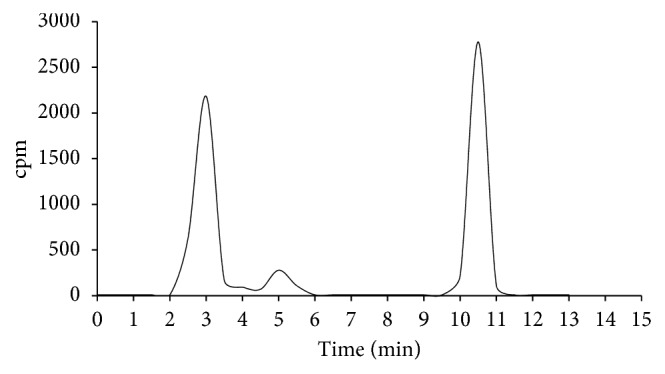
Stability study of ^11^C-VA426 by HPLC analysis, in the plasma of healthy mice (*n* = 3), 10 min p.i.

**Figure 12:**
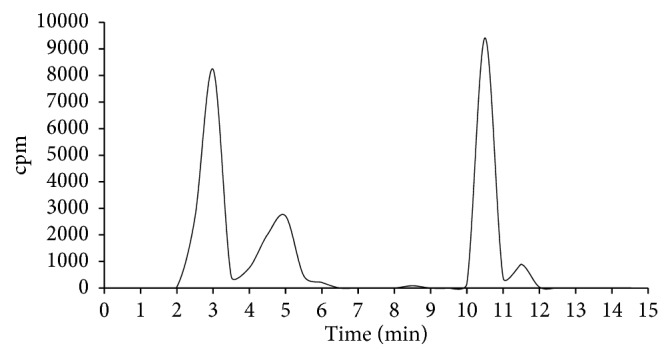
Stability study of ^11^C-VA426 by HPLC analysis, in the liver of healthy mice (*n* = 3), 10 min p.i.

Tracer metabolism could explain the large variability of data observed in biodistribution experiments, as well as the lack of selectivity in LPS experiments; for this reason, we measured the in vivo stability of ^11^C-VA426 in the plasma and liver. Ten minutes after injection, the radioactivity concentration corresponding to the parent compound (10–11 min of retention time) was 47.1% and 34.9% of the total activity in the plasma and liver, respectively (Figures 11 and 12). Plasma extracts showed the presence of two radioactive metabolites more hydrophilic than ^11^C-VA426 (retention times: 3 and 5 min) that accounted for 45.1% and 6.6% of total radioactivity, respectively. Meanwhile in the liver, a third metabolite appeared with less hydrophilicity compared to the ^11^C-VA426 (11.5 min of retention time). The three metabolites reached 39.8%, 21.6%, and 3.3%, respectively.

## 4. Discussion

COX-2 [29] is the inducible isoform of the cyclooxygenase enzyme family (COX-1 and COX-2), which is involved in the development and progression of the inflammatory response, and its frequent overexpression in a variety of human cancers has made it an important drug target for cancer treatment [7, 30, 31]. In this paper, a new potential tracer for in vivo COX-2 monitoring by PET imaging has been developed, exploring its synthesis and radiolabelling and performing a preliminary in vivo evaluation in rodent models. A number of PET and SPECT radiotracers for COX-2 imaging have been synthetized with different radionuclides, including ^18^F and ^11^C, and a restricted group of which was evaluated in the preclinical setting [18, 32]. The ^18^F is often introduced by nucleophilic substitution, and the main problem of ^18^F-labelled compounds is represented by the instability of radionuclide with the consequent defluorination and increase of unspecific signal in animals bones during PET imaging [24]. The introduction of ^11^C in the molecules is made by using [^11^C]methyl iodide as well as [^11^C]methyl triflate. The latter often results to be the best option in terms of yield and purity, and although ^11^C-labelled compounds might be subjected to hepatobiliary modifications, the short half-life of radionuclide certainly represents an advantage from the radioprotection point of view [33]. In general, most of the reported radiotracers failed to visualize COX-2 in vivo due to many limitations, including low metabolic stability, insufficient potency and specificity for COX-2, the lack of suitable preclinical models, and a high nonspecific binding in blood and to other targets [16].

In this work, we focused on the optimization and validation of a fast and completely automated method for the production of the radiolabelled COX-2 selective ligand ^11^C-VA426, which displayed high yields and a good molar activity, therefore representing a promising in vivo imaging agent. In particular, several aspects of [^11^C]CH_3_I production have been addressed and improved to increase the radiochemical yield and the molar activity. In order to exclude water and organic contaminants, like [^12^C]CO_2_, which may reduce molar activity, a target gas mixture containing high purity N_60_ nitrogen and oxygen (99.9999%) was chosen. For the same purposes, hydrogen and helium employed in the synthesis process were passed through gas-purifier traps before using while anhydrous DMSO was further dried over 4 Å molecular sieves. Among tested bases, *t*BuOK resulted to be the most efficient for the deprotonation of the VA425 hydroxyl group, even of a stronger base as NaH. This behaviour is probably due to the nature of NaH that is employed as 60% dispersion in mineral oil that is insoluble in the polar solvent (DMSO) and therefore forms such a “protective pellicle” on the hydride surface. Actually, in classical synthetic procedures, to remove the mineral oil from NaH 60% and then improve its reactivity, this dispersion is rinsed with anhydrous pentane. In this automated synthesis, this protocol should be avoided to reduce loss and/or pollution of the starting material. However, the radiochemical purity observed was always >95%, and both radiochemical yield (15 ± 2%) and molar activity (range 37–148 GBq/*μ*mol) were satisfactory, in about 40 minutes of radiosynthesis.

In the second part of the study, we explored the use of ^11^C-VA426 for noninvasive monitoring of COX-2 distribution, by PET imaging, of potential interest also for the detection of its functional targets, with particular attention in the brain where distribution areas remained unclarified [3]. Our ex vivo biodistribution data in healthy SD rats showed that ^11^C-VA426 maximum uptake was reached at 10 minutes after injection in the kidney, lung, and heart regions of COX-2 moderate expression, but it was cleared thereafter, also in basal conditions [28]. In the liver and intestine, radioactivity concentration remained stable until 60 minutes. Also in the brain, radioactivity picked at 10 minutes p.i., but then rapidly cleared. A slight but general reduction of ^11^C-VA426 uptake was observed after celecoxib preadministration. However, brain distribution data indicated a good radioactivity penetration through the BBB. For this reason, we performed cerebral uptake studies in rats after neuroinflammation induced, by monolateral intrastriatal injection of LPS (PBS contralateral injection, as control), in order to detect a possible radiotracer increase in LPS-ipsilateral compared to the healthy contralateral hemisphere. Results of the study did not evidence any significant difference between the two hemispheres, at both time points examined (1 and 12 days). ^11^C-VA426 suitability was further investigated in mice. As shown in rats, the maximum uptake of radioactivity was observed 10 minutes after injection, confirming previous biodistribution data observed in rats, although the rate of accumulation and clearance was faster and present also in the liver and intestine. In the explorative PET kinetic study performed on two healthy mice (celecoxib or vehicle pre-njected), preadministration of celecoxib (10 mg/kg) showed a reduction of radioactivity concentration especially in intestine areas. Since COX-2 is an inducible enzyme, competition studies were performed six hours after peripheral inflammation induction (LPS intraperitoneal injection). Celecoxib preadministration reduced radioactivity concentration in all organs examined including blood and plasma, indicating that celecoxib administration modified the kinetics of radioactivity distribution in a COX-2 expression independent manner. To understand these results, we evaluated in vivo stability at the time of its maximum uptake (10 minutes after ^11^C-VA426 injection) in the plasma and liver. Results of the analysis showed that approximately over 50% of radioactivity was due to radioactive metabolites, indicating that ^11^C-VA426 is unstable in vivo, thus precluding a further development of the radiopharmaceutical.

## 5. Conclusion

These findings indicate that despite the promising radiolabelling results, ^11^C-VA426 is not suitable as a PET imaging tracer. Further studies are needed in order to improve the in vivo stability of ^11^C-VA426, and to this aim, the methoxyalkyl chain of molecule will be attentively modified.

## Figures and Tables

**Table 1:** Radiosynthesis summary of ^11^C-VA426.

VA425	DMSO	Base^a^	Amount of base	Rad. yield^b^ (%)	SA^c^	*N* ^d^
3.2 mg (8.6 *μ*mol)	150 *μ*l	*t*BuOK	1.5 mg (13 *μ*mol)	15 ± 2	37–148 GBq/*μ*mol	20
2 mg (5.4 *μ*mol)	200 *μ*l	NaH 60%	2 mg (50 *μ*mol)	7 ± 2	37–111 GBq/*μ*mol	20
2 mg (5.4 *μ*mol)	200 *μ*l	NaOH 1N	2 mg (2 *μ*mol)	5 ± 2	37–74 GBq/*μ*mol	5

^a^Influence of the base. ^b^Radiochemical yield (n.d.c. from [^11^C]MeOTf). ^c^Molar activity. ^d^Number of runs. The reaction was carried out at 80°C for 2 minutes.

## Data Availability

The data used to support the finding of this study are available from the corresponding author upon reasonable request.
